# Predictability of clinical outcomes after external beam radiotherapy for hepatocellular carcinoma according to tumor marker dynamics

**DOI:** 10.1371/journal.pone.0323450

**Published:** 2025-05-20

**Authors:** Sunmin Park, Chai Hong Rim, Young Kul Jung, Hyung Joon Yim, Hwan Hoon Chung, Won Sup Yoon

**Affiliations:** 1 Department of Radiation Oncology, Ansan Hospital, College of Medicine, Korea University, Ansan, Gyeong-gi, Republic of Korea; 2 Department of Internal Medicine, Ansan Hospital, College of Medicine, Korea University, Ansan, Gyeong-gi, Republic of Korea; 3 Department of Radiology, Ansan Hospital, College of Medicine, Korea University, Ansan, Gyeong-gi, Republic of Korea; Cincinnati Children’s Hospital Medical Center, UNITED STATES OF AMERICA

## Abstract

Signal changes after high dose irradiation on MRI make it difficult to assess the therapeutic response of hepatocellular carcinoma (HCC). To overcome the limitation of imaging work-up, our study predicted clinical outcomes through tumor marker dynamics in HCC after external beam radiotherapy (EBRT). As a single-center retrospective study, those who underwent conventional fractionated EBRT for viable HCC from 2010 to 2021 were analyzed. Patients with elevated tumor markers of AFP ≥ 10 ng/ml or PIVKA-II ≥ 30 mAU/ml before EBRT were enrolled. Remission of AFP, PIVKA-II, and MoRAL score (=11*√PIVKA-II + 2*√AFP) from pre-EBRT to post-EBRT at 1 month and 3 months was examined. For 1-year and 2-years OS, variables of tumor markers were examined using the receiver operation characteristics (ROC). Multivariate analyses of Cox-regression for OS were conducted. Among 111 patients, 34 patients were estimated to survive more than 2-years. In multivariate analyses for OS, tumor number (*P* = 0.004), portal vein tumor thrombus (*P* = 0.004), and Barcelona liver cancer staging (*P* < 0.001) were found to be significant. For 2-years OS, the degree of AFP remission at 3 months (rAFP_3M) had an AUC of 0.852 (95% CI: 0.758–0.946, *P* < 0.001), a sensitivity of 85.5%, and a specificity of 82.6% with a cut-off value of 3.7%. MoRAL score at 3 months (MoRAL_3M) had an AUC of 0.814 (95% CI: 0.728–0.900, *P* = 0.000), a sensitivity of 76.5%, and a specificity of 77.8% with a cut-off value of 111.64. In new multivariate analyses including the above significant factors plus either rAFP_3M or MoRAL_3M, rAFP_3M (*P* < 0.001) and MoRAL_3M (*P* < 0.001) were found to be independent prognostic factors in each model. This study confirmed the importance of the changed tumor marker after EBRT rather than the baseline value. Dynamic change of AFP and MoRAL score at post-EBRT 3 months could be recommended as potential indicators for clinical outcomes.

## Introduction

Hepatocellular carcinoma (HCC) is common in endemic area of hepatitis B virus (HBV) and C virus (HCV) and the prevalence and mortality of HCC still remain high in Asia-Pacific region [1]. However, overall survival (OS) of patients with HCC has increased from 21.7% in 1999–2005 to 42.4% in 2011–2019 in Korea thanks to advancement of diagnostic methods, diversification of treatment methods, and introduction of systematic targeted chemotherapy [2].

External beam radiotherapy (EBRT) has not been considered in major therapeutic methods in European Association for the study of the Liver (EASL) guideline for HCC [3]. However, EBRT has gradually raised indications in Asian endemic region. Especially, the Korean Liver Cancer Study Group (KLCSG) practice guideline suggests standard and second options of EBRT in various clinical situations based on a modified Union for International Cancer Control (mUICC) staging [4]. It describes modern delivery methods to make therapeutic ratio better and achieve synergic effects with other therapeutic methods such as trans-arterial chemoembolization (TACE), hepatic artery infusion chemotherapy (HAIC), and targeted therapies [5]. A randomized trial has demonstrated that EBRT prior to or after TACE can improve survival in a cohort with portal vein tumor thrombus (PVTT) [6]. Both cohort studies in Korea and Taiwan have shown better clinical outcomes of EBRT plus sorafenib in locally advanced HCC [7,8].

The conventional EBRT does not present immediate effect after radiation. It needs sufficient time to induce cell death. Generally, it is expected to take about 3–4 months to reach maximum response. Therefore, to determine outcomes of definite EBRT, imaging methods are recommended after sufficient time has passed. In HCC, modified Response Evaluation Criteria in Solid Tumors (mRECIST) are commonly used to differentiate a fibrotic residual lesion and a viable tumor. The objective response based on mRECIST is assessed as an independent predictor of survival in meta-analysis [9]. However, in the case of EBRT, the liver receiving high doses of radiation around the main target can cause signal changes in magnetic resonance imaging (MRI), which is a limiting factor in precise response evaluation [10].

Among tumor markers, serum α-fetoprotein (AFP) and prothrombin induced by vitamin K absence-II (PIVKA-II) are known as useful factors for the diagnosis and surveillance in HCC [11]. However, the relevance of AFP and PIVKA-II to the response after EBRT has not been closely studied. Thus, the purpose of this study was to examine the relationship between tumor marker changes and survival using clinical outcomes after EBRT in HCC. Specifically, this study aimed to identify useful factors among tumor markers and determine the appropriate timing of measurement.

## Materials and methods

### Eligibility

This study retrospectively collected medical records of patients who received EBRT for treating HCC in single institution of Korea University Ansan Hospital from September 2010 to July 2021. Subjects of this study were patients whose tumor marker levels were elevated based on either AFP ≥ 10 ng/ml or PIVKA-II ≥ 30 mAU/ml before EBRT. The remaining inclusion criteria were as follows: (1) HCC was diagnosed based on the KLCSG practice guideline at that time; (2) an Eastern Cooperative Oncology Group (ECOG) performance score of 0–2; (3) Child-Pugh (CP) class A or B; and (4) EBRT was performed on the liver over a biological effective dose (BED) of 40 Gy_10_ when the α/β ratio was assumed to be 10 Gy. Exclusion criteria were as follows: (1) patients who missed their follow-up evaluation at the end of EBRT; (2) stereotactic radiotherapy (SBRT) was done with < 10 fractions; (3) disseminated regional or distant metastases (the number of metastases ≥ 5 on ≥ 3 organs); and (4) inferior vena cava invasion or heart invasion. This study was approved by the institutional review board (IRB) of Korea University Ansan Hospital (approval number: 2023AS0275). Written informed consent was waived by the IRB due to the retrospective nature of this study. Our institution allowed access to patient’s clinical data for research purposes from September 21, 2023.

### Radiotherapy

For simulation, computerized tomography (CT) images with a thickness of 2 mm or 3 mm were acquired using Big Bore CT simulation (Philips Medical System, The Netherlands). CT images were taken under free breathing with respiratory gating. In addition, a fixation device or abdominal compression equipment was applied in many cases. After images were transported to an Eclipse radiotherapy planning system (Varian Medical system, Palo Alto, CA, USA), MRI was registered to simulation CT images to draw gross target volume (GTV). Referring to images of respiratory gating, GTV was extended to internal target volume (ITV). Then a 3–5 mm was added from ITV to make planning target volume (PTV). To make optimal RT planning for EBRT, the radiation dose to liver was constrained to meet the KLCSG practice guideline if possible. In addition, the dose schedule of EBRT was determined considering the spinal cord dose as well as the bowel dose including duodenum.

### Patients information

Various kinds of clinical information were examined. Personal information (such as age, sex, and hepatitis), clinical information (such as CP score, liver function tests, and disease status), and treatment information regarding EBRT and other concurrent or sequential therapies were obtained. Overall survival (OS) was calculated from the start day of EBRT date to the date of death or available follow-up. Disease free survival (DFS) used the day of any recurrence from the same start day. This study evaluated the possibility of 1-year survival (OS-1Y) and 2-year survival (OS-2Y). For OS-1Y and OS-2Y, patients whose disease were disseminated and performance had worsened with ECOG performance ≥ 3 were regarded as death at the last follow-up.

### Tumor marker analyses

AFP and PIVKA-II had maximal values to be able to measure in actual clinics. When the measured value exceeded the maximum value, the maximum value was replaced by the measured value to decrease missing data. Another factor of MoRAL score (= 11*√PIVKA-II + 2*√AFP) that combined AFP and PIVKA-II was calculated. Tumor markers according to three measurement periods at pre-treatment, 1 month after EBRT, and 3 months after EBRT were used. If the combined therapy was done within 4 weeks of EBRT, tumor markers at pretreatment used values before the combined therapy. Because this study was retrospective, a broad window period was applied as likely as ± 2 weeks and ± 1 month for 1 month and 3 months measurement periods, respectively. If the tumor marker decreased to within the normal range (AFP < 7.5 ng/ml or PIVKA < 20 mAU/ml during follow-up, it was regarded as complete response. The followings factors were used as test variables. In addition to AFP, PIVKA-II, and MoRAL were applied.

AFP_Pre: raw value of AFP at pre-treatment
AFP_1M: raw value of AFP at 1 month after EBRT
AFP_3M: raw value of AFP at 3 months after EBRT
rAFP_1M: 100*(AFP_1M/ AFP_pre)
rAFP_3M: 100*(AFP_3M/ AFP_pre)

### Statistics

For OS-1Y and OS-2Y, variables of tumor markers were examined using the receiver operation characteristics (ROC). According to the best specificity and sensitivity, the cut-off value was determined. New models of multivariate analyses adding significant tumor marker variable were then suggested. The OS was evaluated by the Kaplan-Meier method. Log-rank tests were used for comparing survival differences in terms of various factors within subgroups. Prognostic factors with a *P*-value of less than 0.1 in the univariate analyses were entered into the multivariate analyses of Cox-regression for OS using the backward elimination method. A *P*-value of less than 0.05 was considered significant. The Statistical Package for the Social Sciences (SPSS) version 21.0 (IBM Corp., Armonk, NY, USA) was used to perform all statistical analyses.

## Results

### Patient characteristics

A total of 111 patients satisfied the inclusion criteria. They were enrolled in this study. Their median age was 58 years. Most (90.1%) of them were males. Those who had HBV and HCV accounted for 65.8% and 10%, respectively. Although at least one of the three liver function tests was elevated in many cases, the CP class was stable with A. While there were 40 cases in which surrounding HCC and portal vein tumor thrombus (PVTT) were simultaneously targeted as PTV, only PVTT was included in 21 cases. Various EBRT schedules were applied, with a median BED of 58.5 Gy_10_. AFP and PIVKA-II elevated in 86 and 104 patients, respectively.(Table 1)

**Table 1:** Patient characteristics.

		Entire (N = 111)	AFP (≥10ng/ml) (N = 86)	PIVKA-II (≥30mAU/ml) (N = 104)
Age (years)	Median (Range)	58 (33-87)	56.5 (33-82)	58 (33-87)
	<65: ≥ 65	77: 34	64: 22	71: 33
Sex	Male: Female	101: 10	77: 9	94: 10
Child-Pugh score	5: 6: ≥ 7	75: 20: 16	57: 18: 11	69: 19: 16
Albumin (g/ml)	≥3.5: < 3.5	100: 11	80: 6	93: 11
Bilirubin (mg/ml)	<1.5: ≥ 1.5	86: 25	66: 20	81: 23
Liver function test	Normal: Elevation	27: 84	17: 69	25: 79
Location	Unilateral: Bilateral	67: 44	47: 39	62: 42
Tumor size (cm)	≤5: > 5	34: 77	24: 62	30: 74
Lesions number	1: 2-4: ≥ 5	27: 55: 29	19: 40: 27	24: 52: 28
PVTT	No: Yes	50: 61	32: 54	47: 57
AFP (ng/ml)	<500: ≥ 500	43: 68	18: 68	42: 62
PIVKA-II (mAU/ml)	<2000: ≥ 2000: unknown	52: 57: 2	38: 46: 2	47: 57
BCLC	A: B: C	23: 25: 63	14: 18: 54	20: 25: 59
Combined therapy	TACE: HAIC: Systemic: RFA: None	74: 17: 6: 1: 13	60: 15: 5: 1: 5	69: 17: 5: 1: 12
BED (Gy_10_)	Median (Range)	58.5 (46.8 - 119.0)	60.0 (46.8 - 119.0)	58.0 (46.8 - 119.0)

PVTT; portal vein tumor thrombus, BCLC; Barcelona Clinic Liver Cancer staging, TACE; transarterial chemembolization, HAIC; hepatic artery chemoinfusion, RFA; radiofrequency ablation, BED; biological effective dose assuming α/β ratio = 10

### Survival analyses

The disease was progressed in 89 patients after EBRT. One-year DFS was 25.0%. Numbers of patients included in OS-1Y and OS-2Y after EBRT were 54 and 34, respectively. In multivariate analyses for OS, several prognostic factors including sex (*P* = 0.040), CP class (*P* = 0.007), elevated liver function tests (*P* = 0.008), lesions number (*P* = 0.004), PVTT (*P* = 0.004), and BCLC stage (*P* < 0.001) were examined.(Table 2) Stratified tumor markers based on cut-off values of AFP (500 ng/ml) and PIVKA-II (2000 mAU/ml) were significant in log-rank test. However, they were eliminated as main factors for OS in multivariate analyses. The combined therapies during the interval at 1 month to 3 months to measure tumor markers were taken in 32(28.8%) case, however this factor did not affect OS.

**Table 2:** Univariate and multivariate analyses of overall survival in entire cohort.

		Log-Rank		Cox-regression		
	Subgroup	Median survival	P value	HR	95% CI	P value
Age (years)	<65	10.2	0.093			
	≥65	18.5				
Sex	Male	11.4	0.023	2.875	1.045 - 7.905	0.041
	Female	41.9				
Child-Pugh	A	16.3	0.024	2.343	1.262 - 4.353	0.007
	B	7.4				
Liver function test	Normal	31.2	0.001	2.147	1.217 - 3.785	0.008
	Elevation	9.7				
Tumor size (cm)	≤5	17.7	0.026			
	>5	10.2				
Lesions number	1	17.3	ref. 1			ref. 1
	2,3,4	15.4	0.054	1.823	0.958 - 3.469	0.067
	≥5	9.2	0.002	3.471	1.630 - 7.390	0.001
AFP (ng/ml)	<500	20.1	0.022			
	≥500	9.7				
PIVKA-II (AU/ml)	<2000	20.1	0.044			
	≥2000	9.8				
MoRAL score	<500	20.1	0.002			
	≥500	9.7				
PVTT	No	30.7	<0.001	0.103	0.022 -.481	0.004
	Yes	9.6				
BCLC	A	48.0	ref. A			
	B	27.5	0.016	0.946	0.396 - 2.259	0.900
	C	9.2	<0.001	22.990	4.391 - 120.361	<0.001

PVTT; portal vein tumor thrombus, BCLC; Barcelona Clinic Liver Cancer staging.

### Optimal tumor markers

**Fig 1:**
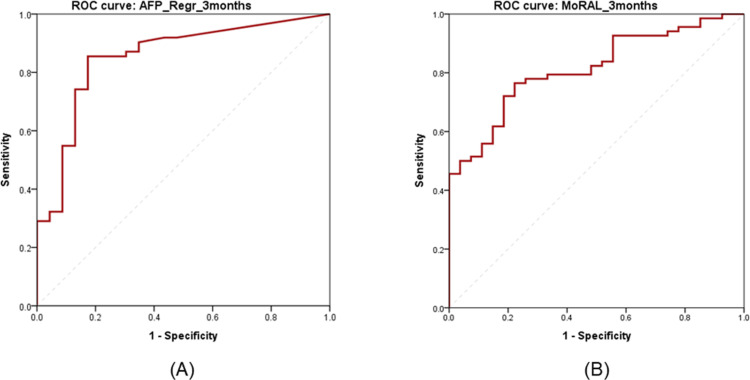
ROC curve for OS-2Y. (A) AUC of rAFP_3M was 0.852 (95% CI: 0.758–0.946, *P* < 0.001). (B) AUC of MoRAL_3M were 0.814 (95% CI: 0.728–0.900, *P* < 0.001).

For OS-1Y and OS-2Y, the predictive power of tumor marker was evaluated.(Table 3) In general, the area under curve (AUC) of OS-2Y was higher than that of OS-1Y in each factor of tumor marker. Regression variables showed that 3 months outcomes tended to be superior to 1-month outcomes, implying the importance of tumor marker after achieving sufficient treatment effects. For OS-2Y, rAFP_3M with an AUC of 0.852 (95% CI: 0.758–0.946, *P* < 0.001) was the best. When the cut-off value was 3.7%, the sensitivity and specificity were 85.5% and 82.6%, respectively.(Fig 1) For OS-2Y, MoRAL_3M had an AUC of 0.814 (95% CI: 0.728–0.900, *P* < 0.001). When the cut-off value was 111.64, the sensitivity and specificity were 76.5% and 77.8%, respectively.(Fig 1)

**Table 3:** Univariate analyses for tumor marker threshold and ROC curve.

	D/S/M	AUC	95% CI	P value	Cut-off	Sensitivity	Specificity
OS_1Y							
rAFP_1M	47/38/26	0.699	0.588 - 0.811	0.002	29.6	0.681	0.632
rAFP_3M	47/38/26	0.798	0.703 - 0.893	<0.001	14.3	0.809	0.711
rPIVKA_1M	49/48/14	0.669	0.561 - 0.776	0.004	16.3	0.653	0.646
rPIVKA_3M	46/45/20	0.771	0.675 - 0.868	<0.001	16.7	0.717	0.711
rMoRAL_1M	52/49/10	0.713	0.613 - 0.814	<0.001	57.2	0.673	0.653
rMoRAL_3M	50/45/16	0.78	0.687 - 0.872	<0.001	53.2	0.720	0.689
AFP_Pre	48/38/25	0.508	0.384 - 0.633	0.893	551.0	0.583	0.474
AFP_1M	56/52/3	0.683	0.581 - 0.785	0.001	74.2	0.661	0.731
AFP_3M	56/54/1	0.758	0.668 - 0.849	<0.001	38.8	0.732	0.741
PIVKA_Pre	56/53/2	0.606	0.499 - 0.712	0.057	1097.0	0.607	0.604
PIVKA_1M	53/50/8	0.7	0.597 - 0.802	<0.001	95.0	0.679	0.64
PIVKA_3M	49/46/16	0.769	0.674 - 0.865	<0.001	146.5	0.735	0.696
MoRAL_Pre	56/53/2	0.602	0.496 - 0.709	0.065	549.0	0.571	0.660
MoRAL_1M	53/50/8	0.715	0.612 - 0.816	<0.001	150.0	0.660	0.640
MoRAL_3M	49/46/16	0.781	0.686 - 0.875	<0.001	176.0	0.735	0.739
OS_2Y							
rAFP_1M	62/23/26	0.765	0.649 - 0.882	<0.001	9.6	0.790	0.652
rAFP_3M	62/23/26	0.852	0.758 - 0.946	<0.001	3.7	0.855	0.826
rPIVKA_1M	66/31/14	0.693	0.582 - 0.805	0.002	9.6	0.697	0.677
rPIVKA_3M	64/27/20	0.745	0.636 - 0.854	<0.001	6.7	0.734	0.704
rMoRAL_1M	70/31/10	0.684	0.574 - 0.795	0.003	49.4	0.614	0.613
rMoRAL_3M	68/27/16	0.702	0.587 - 0.817	0.002	48.0	0.632	0.593
AFP_Pre	63/23/25	0.504	0.361 - 0.646	0.957	551.0	0.571	0.478
AFP_1M	74/34/3	0.734	0.631 - 0.836	<0.001	18.3	0.743	0.676
AFP_3M	76/34/1	0.785	0.697 - 0.873	<0.001	18.4	0.724	0.735
PIVKA_Pre	75/34/2	0.642	0.533 - 0.751	0.018	740.0	0.613	0.618
PIVKA_1M	71/32/8	0.741	0.646 - 0.837	<0.001	63.5	0.704	0.688
PIVKA_3M	68/27/16	0.765	0.668 - 0.862	<0.001	72.5	0.721	0.704
MoRAL_Pre	75/34/2	0.684	0.580 - 0.788	0.002	448.0	0.600	0.647
MoRAL_1M	72/31/8	0.786	0.699 - 0.874	<0.001	114.2	0.750	0.710
MoRAL_3M	68/27/16	0.814	0.728 - 0.900	<0.001	111.6	0.765	0.778

D; death, S; survival, M: missing, AUC; area under curve, OS_1(2)Y; 1(2)-year OS, rAFP_1M(3M): response of AFP from pretreatment to 1(3) month after external beam radiotherapy, AFP_1(3)M; raw value of AFP at 1(3) month after external beam radiotherapy.

### Survival effect of tumor markers

**Fig 2:**
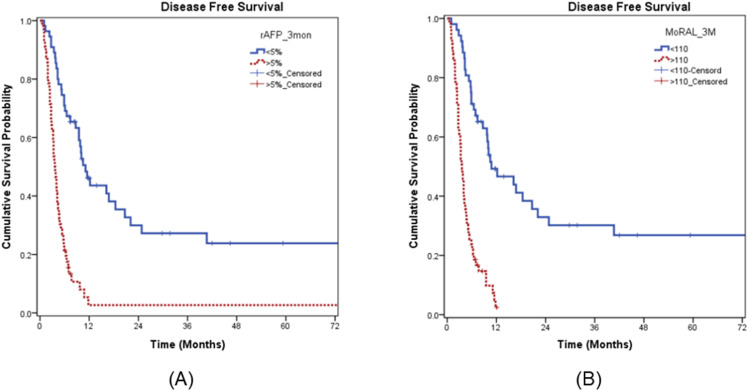
DFS after EBRT. (A) On the basis of the cut-off value of 5% for rAFP_3M, median survival was different as 11.1 vs. 3.6 months. (B) On the basis of the cut-off value of 110 for MoRAL_3M, median survival was different as 10.8 vs. 3.6 months.

**Fig 3:**
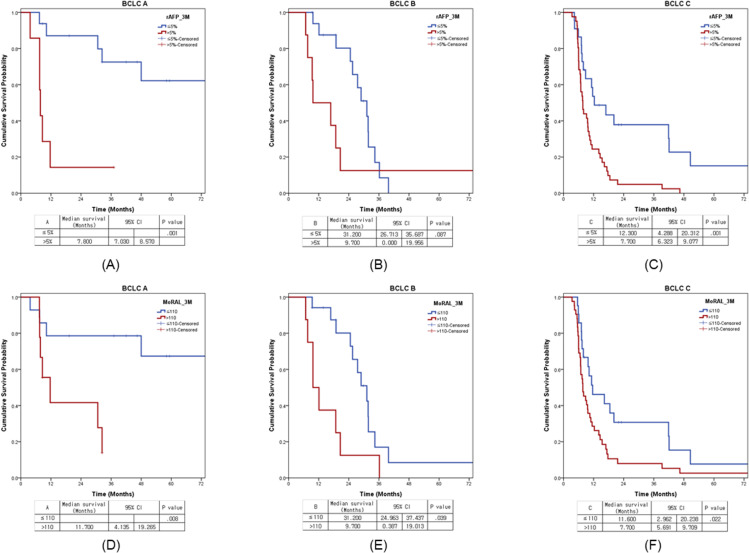
Survival difference in BCLC stage in terms of the dynamic change of tumor marker. **The survival curves of rAFP_3M (≤5% (blue line) and >5% (red line)) were presented in terms of BCLC stage of A (A), B (B) and C (C) panels. The survival curves of MoRAL_3M (≤110 (blue line) and >110 (red line) were presented in terms of BCLC stage of A (D), B (E) and C (F)**.

**Fig 4:**
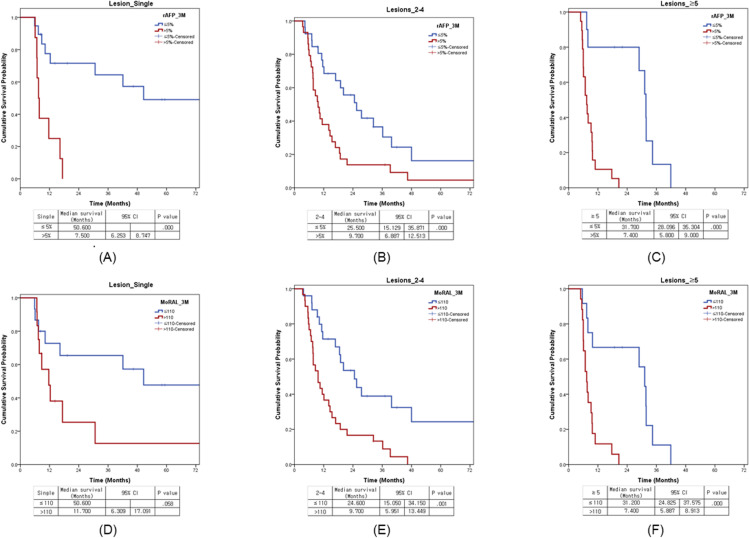
Survival difference in lesions number in terms of the dynamic change of tumor marker. **The survival curves of rAFP_3M (≤5% (blue line) and >5% (red line)) were presented in terms of lesion number for single (A), 2-4 (B) and ≥ 5 (C). The survival curves of MoRAL_3M (≤110 (blue line) and >110 (red line)) were presented in terms of lesion number for single (D), 2-4 (E) and ≥ 5 (F)**.

After creating a stratified group based on rAFP_3M of 5% and MoRAL_3M of 110, when each factor was added and analyzed in the above-mentioned multivariate analysis, rAFP_3M and MoRAL_3M were evaluated as main prognostic factors in each model.(Table 4) For DFS, rAFP_3M of 5% (median 11.1 months vs. 3.6 months, P < 0.001) and MoRAL_3M of 110 (median 10.8 months vs. 3.6 months, P < 0.001) were significant.(Fig 2) In sub-groups of BCLC C stage, median OS were 12.3 months and 7.7 months according to cut-off value 5% of rAFP_3M (*P* = 0.001), and 11.6 months and 7.7 months according to cut-off value 110 of MoRAL_3M (*P* = 0.022). (Fig 3) In another sub-groups of lesions number_2–4, median OSs were 25.5 months and 9.7 months according to cut-off value 5% of rAFP_3M (*P* < 0.001) and 24.6 months and 9.7 months according to cut-off value 110 of MoRAL_3M (*P* = 0.001).(Fig 4)

**Table 4:** Multivariate analyses when MoRAL_3M and rAFP_3M are added to existing clinical factors.

	P value	HR	95% CI
rAFP_3M (cut-off 5%)			
Child-Pugh B	0.024	2.022	1.095 - 3.733
Lesions number	ref. 1		
2–4	0.914	1.040	0.512 - 2.111
≥5	0.094	1.982	0.890 - 4.410
PIVKA-II ≥ 2000	0.048	1.589	1.004 - 2.514
PVTT	0.000	.041	0.008 -.206
BCLC	ref. A		
B	0.508	1.345	0.559 - 3.234
C	<0.001	52.528	9.545 - 289.067
rAFP_3M	<0.001	3.927	2.274 - 6.782
MoRAL_3M (cut-off 110)			
Child-Pugh B	0.033	1.948	1.056 - 3.594
Lesions number	ref. 1		
2–4	0.151	1.611	0.841 - 3.086
≥5	0.003	3.103	1.458 - 6.602
PVTT	0.003	.094	0.020 - 0.440
BCLC	ref. A		
B	0.330	1.519	0.655 - 3.520
C	<0.001	26.873	5.154 - 140.107
MoRAL_3M	<0.001	2.643	1.627 - 4.292

PVTT; portal vein tumor thrombus, BCLC; Barcelona Clinic Liver Cancer staging, MoRAL-3M; raw value of MoRA at 3 month after external beam radiotherapy, rAFP_3M: response of AFP from pretreatment to 3 month after external beam radiotherapy.

## Discussion

HCC is frequently progressed as shown in DFS of this study. Therefore, evaluation of current treatment method should be made at an appropriate time and the next-line treatment should be prepared accordingly. Generally, the response of solid tumors would be determined though imaging methods after 6–8 weeks of the end of conventional EBRT [12]. However, there are no HCC specific principles of care during or immediate after definite EBRT. Tumor markers of AFP and PIVKA-II are expected to play additional role along with imaging methods in HCC. This study tried to understand the status of HCC after EBRT and determine the value of tumor markers through careful observation of AFP and PIVKA-II.

The cohort consisted of patients with stable liver function of CP A and B. However, advanced HCC was more than half with PVTT. Most of the patients underwent EBRT in combined with other treatments, therefore our results were not specific to EBRT. Our endpoint was presented as OS because tumor markers are not only associated with the local response, but also the new occurrence of regional or distant metastases. Although it was a retrospective study, follow-up examination was performed about once a month immediately after EBRT and tumor markers were measured within the valid period desired in this study. Therefore, bias due to missing data could be minimal. Because the OS of HCC was affected by not only tumor status but also the preservation of liver function, detailed survival show some differences depending on the quality of supportive care. Therefore, we applied the possibility of OS to determine the cut-off value of tumor marker. Main results of this study showed that tumor markers were able to predict mid-term survival (2-year OS) better than short-term (1-year OS) and post-treatment values at 3 months after EBRT were more effective than pre-treatment value. Both rAFP_3M and MoRAL_3M, the main factors of our study, would need to be noted more in future studies. They were also effective in multivariate analysis for OS. They showed importance in prognosis for subgroups of BCLC C stage and lesions number of 2–4, which were independent prognostic factors for OS. Thus, they are expected to be useful for evaluating response to EBRT along with imaging methods

Baseline AFP and PIVKA-II are well known prognostic factors. Recently, the importance of monitoring tumor markers after definite treatment has emerged. However, it is still difficult to draw a clear conclusion because the degree of AFP reduction compared to the baseline value varies. In addition, the timing to assess the response might vary depending on treatment methods. Since most studies were retrospective cohort studies, they have limitations in reaching a consensus. In Chinese retrospective studies, a sharp-falling of AFP was significant for predicting OS after hepatectomy (a high preoperative AFP value > 200 ng/mL, declining rapidly below 25 ng/mL in the second or third follow-up within four months) and TACE (first AFP measuring ≥ 316 ng/mL, declining toward at least < 100 ng/mL within 4 months) in intermediate staged HCC [13,14]. In a cohort liver transplantation after TACE, decreases of AFP after TACE and initial AFP were related to complete tumor necrosis in histologic examination [15]. In an Indian study, a reduction of AFP < 30% could differentiate responders from non-responders with a sensitivity of 70%, a specificity of 68%, and an AUC of 0.74 after locoregional therapy [16]. In a study applying SBRT, PIVKA-II value < 25 mAU/ml after SBRT was related to DFS [17]. In a cohort receiving chemotherapy of atezolizumab plus bevacizumab, 30% reduction was an independent predictor of objective response (odds ratio: 5.517; *p* = 0.003) in HCC with baseline AFP ≥ 20 ng/mL [18]. In our study, the cut-off value was relatively stricter than previous studies. There were many cases (33.3%) where initial AFP was higher than 1000 ng/mL and moderate cases (15.3%) where AFP decreased below the normal range at 3 months after EBRT. These factors might have influenced the strict cut-off value of this study.

MoRAL score was suggested through the combination of AFP and PIVKA-II. It was efficient to predict refractory HCC to TACE in an intermediate stage. The case where the baseline was ≥ 89.5 and the following MoRAL after TACE was higher than the baseline was judged as a resistant group [19]. In early stage HCC, baseline MoRAL score > 68 was significant as a predictive factor of tumor recurrence after radiofrequency ablation [20]. In living donor liver transplantation, a high MoRAL score > 314.8 was significant as a predictive factor of recurrence and OS in the beyond Milan Criteria sub-cohort [21]. In this study, we confirmed that the change of median MoRAL score from 339.7 of pre-EBRT to 192.0 of 3month after EBRT in EBRT alone group. We thought that it seemed to be useful in patients with only one of AFP and PIVKA-II elevated and suggested the cut-off value of 110. The reference score of MoRAL related to the prognosis needs further studies. Another laboratory score using ALBI score and AFP had also been attempted to evaluate the prognosis after TACE [22].

Our study revealed that the evaluation at 3 months after EBRT was superior to that at 1 month. However, we could not conclude whether the specific time point after EBRT was better. Although one study showed that the response of AFP after EBRT was continued up to 9 months, it was a single institutional retrospective study with a small number of patients [23]. A few studies have also shown that the timing of evaluation after SBRT in early staged lung cancer requires sufficient period [24]. However, in advanced HCC, intrahepatic recurrence out of target volume of EBRT is common. Therefore, it seems that the response assessment does not need to be delayed too much. About 3 months is considered an appropriate evaluation time.

Radiomics is often mentioned in relation to prognosis in HCC. In one study performing EBRT for PVTT, a nomogram was made using rad-score from radiomics analyses, clinical factors of sex, CP class, anemia, and dosimetric parameter of mean liver dose. The AUC of 9 months OS was 0.71 (95% CI: 0.63–0.79) [25]. In another studies that performed SBRT for BCLC C, radiomics factor including CP class, tumor size, and BED were found to be important factors to establish nomogram, showing that the AUC of 1-year OS was 0.79 (95% CI: 0.63–0.93) [26]. There was also a study that mentioned Immune parameters such as neutrophil to lymphocyte ratio and systematic immune inflammatory [27]. Lens culinaris agglutinin-reactive fraction of AFP (AFP-L3) and des-gamma-carboxy prothrombin (DCP) as biomarkers with research actively conducted need to be considered in the response after EBRT [28,29].

This study had some limitations. Firstly, the cohort was heterogeneous from early stage to locally advanced stage. EBRT technique had been changed from the era of 3D conformal RT to intensity modulated RT. Accordingly, fraction size increased and treatment period became shorter. To minimize the number of missing data, the window period for tumor markers was set to be slightly wide. Those were regarded as the limitations of this study from a retrospective nature. Secondly, systemic therapy was not commonly included. Although this study excluded metastatic disease, liver direct therapy was preferred to systemic therapy in locally advanced disease. Since many guidelines suggest sorafenib, lenvatinib, atezolizumab plus bevacizumab, and tremelimumab plus durvalumab as standard therapies that are widely used in our institution recently, it is necessary to examine whether rAFP_3M and MoRAL_3M would be useful even in an environment in which EBRT is combined with systemic therapy. Lastly, this study had some assumptions for tumor markers. Criteria for normal values, complete regression, and measurable maximum values are not completely established. Therefore, caution is needed when interpreting our results.

## Conclusions

This study confirmed the importance of the changed tumor marker after EBRT rather than the baseline value. In HCC with elevated tumor markers, the dynamic change of AFP at 3 months after EBRT and MoRAL score at 3 months after EBRT could be recommended as potential indicators. Although population size was limited, those factors showed significance in each subgroup of BCLC stage and tumor number. Optimal measurement time based on the time point of 3 months after EBRT would be validated in follow-up research. If present tumor markers, radiomics and biomarker under development are added, the prognostic evaluation could be more precise.
